# Multifactorial Genetic and Environmental Hedgehog Pathway Disruption Sensitizes Embryos to Alcohol‐Induced Craniofacial Defects

**DOI:** 10.1111/acer.14427

**Published:** 2020-08-30

**Authors:** Joshua L. Everson, Rithik Batchu, Johann K. Eberhart

**Affiliations:** ^1^ From the Department of Molecular Biosciences School of Natural Sciences University of Texas at Austin Austin Texas USA; ^2^ Waggoner Center for Alcohol and Addiction Research School of Pharmacy University of Texas at Austin Austin Texas USA

**Keywords:** Birth Defect, Prenatal Alcohol Exposure, Fetal Alcohol Spectrum Disorders, Gene, Environment Interactions, Craniofacial

## Abstract

**Background:**

Prenatal alcohol exposure (PAE) is perhaps the most common environmental cause of human birth defects. These exposures cause a range of structural and neurological defects, including facial dysmorphologies, collectively known as fetal alcohol spectrum disorders (FASD). While PAE causes FASD, phenotypic outcomes vary widely. It is thought that multifactorial genetic and environmental interactions modify the effects of PAE. However, little is known of the nature of these modifiers. Disruption of the Hedgehog (Hh) signaling pathway has been suggested as a modifier of ethanol teratogenicity. In addition to regulating the morphogenesis of craniofacial tissues commonly disrupted in FASD, a core member of the Hh pathway, Smoothened, is susceptible to modulation by structurally diverse chemicals. These include environmentally prevalent teratogens like piperonyl butoxide (PBO), a synergist found in thousands of pesticide formulations.

**Methods:**

Here, we characterize multifactorial genetic and environmental interactions using a zebrafish model of craniofacial development.

**Results:**

We show that loss of a single allele of *shha* sensitized embryos to both alcohol‐ and PBO‐induced facial defects. Co‐exposure of PBO and alcohol synergized to cause more frequent and severe defects. The effects of this co‐exposure were even more profound in the genetically susceptible *shha* heterozygotes.

**Conclusions:**

Together, these findings shed light on the multifactorial basis of alcohol‐induced craniofacial defects. In addition to further implicating genetic disruption of the Hh pathway in alcohol teratogenicity, our findings suggest that co‐exposure to environmental chemicals that perturb Hh signaling may be important variables in FASD and related craniofacial disorders.

## Graphical abstract

Prenatal alcohol exposure is a major cause of craniofacial birth defects in humans, but outcomes vary widely. Using zebrafish, we tested the hypothesis that genetic predisposition or co‐exposure to additional environmental toxicants can interact with alcohol to worsen craniofacial defects. We identified synergistic interactions between three factors: alcohol, the common pesticide synergist and recently identified Hedgehog pathway inhibitor piperonyl butoxide (PBO), and mutations in the Hedgehog pathway. Together, this work demonstrates multifactorial interactions can exacerbate alcohol‐induced craniofacial defects.

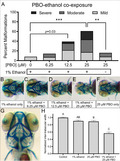

Prenatal alcohol exposure (PAE) is a leading cause of developmental defects, resulting in structural and neurocognitive disorders collectively known as fetal alcohol spectrum disorders (FASD) (Parnell and Chambers, 2019; Riley, Infante and Warren, 2011; Riley et al., 2001; Warren and Foudin, 2001). FASD affect 1 to 8% of children born each year in the United States (May et al., 2014; May et al., 2009; May et al., 2020). It is estimated that 10% of women worldwide consume alcohol during pregnancy (Popova et al., 2017; Popova et al., 2018). The embryo’s period of susceptibility to alcohol spans first, second, and third trimesters in humans, but the most severe craniofacial malformations typically arise from a first trimester exposure. Evidence from mice demonstrates the stereotypic facial abnormalities associated with PAE occur following a gastrulation‐stage (gestational day 7) exposure (Lipinski et al., 2012b; Sulik, 1984). This period of susceptibility is conserved in zebrafish, where analogous defects are observed following gastrulation‐stage exposure (Lovely, Fernandes and Eberhart, 2016; McCarthy et al., 2013; Swartz et al., 2020; Swartz et al., 2014). This critical period is equivalent to approximately 3‐week pregnancy in humans (Sulik, 1984), before most pregnancies are identified. This likely contributes to PAE remaining a continued problem, despite widespread communication of the significant developmental risk posed by alcohol use during pregnancy.

Alcohol causes a wide range of clinical phenotypes, which cannot be fully explained by dose, timing, or duration. That is, the same alcohol exposure that is teratogenic for one embryo may not cause observable dysmorphologies in another (Astley Hemingway et al., 2018). The causes of this variability are poorly understood, but one factor appears to be genetics (Eberhart and Parnell, 2016; Lovely et al., 2017; McCarthy and Eberhart, 2014).

A genetic pathway that has been widely implicated as a modifier of ethanol teratogenicity is the Hedgehog (Hh) signaling pathway. The Hh signaling pathway is a critical regulator of brain and face development in vertebrates (Eberhart et al., 2006; Hu and Marcucio, 2009; Marcucio et al., 2005; Swartz et al., 2012; Wada et al., 2005). Inhibition of the pathway can cause specific brain and face malformations, depending on the developmental stage in which signaling is perturbed (Abramyan, 2019; Chiang et al., 1996; Heyne et al., 2015; Lipinski et al., 2010; Zhang et al., 2006). These defects include holoprosencephaly (HPE), a malformation that is also associated with PAE (Addissie et al., 2020; Cohen, 2006). Studies in mice demonstrate that a gastrulation‐stage ethanol exposure can cause HPE (Hong and Krauss, 2017; Sulik, 1984). In addition, mice with heterozygous mutations in Hh pathway genes (e.g., *Shh*, *Gli2*, and *Cdon*) are sensitized to ethanol‐induced defects (Hong and Krauss, 2012; Kahn et al., 2017; Kietzman et al., 2014). In zebrafish, reductions in Shh sensitize embryos to ocular and neural defects (Burton et al., 2017; Zhang, Anderson and Cole, 2015; Zhang et al., 2014; Zhang, Ojiaku and Cole, 2013). Lastly, Hh signaling has been shown to be disrupted following exposure to ethanol (Li et al., 2007), though whether this effect on the pathway is direct or indirect remains unclear.

In addition to genetic perturbation, Hh signaling can be modulated by environmental chemicals. The transmembrane protein Smoothened (Smo) is essential for Hh signaling and is inhibited by a wide variety of structurally diverse compounds (Chen et al., 2002; Frank‐Kamenetsky et al., 2002; Lipinski and Bushman, 2010). For example, the dietary alkaloids tomatidine, solanidine, and solasodine found in Solanaceae (nightshades) like tomatoes, potatoes, peppers, and eggplant, as well as curcumin in turmeric, can each antagonize Smo and block pathway activity (Elamin et al., 2010; Lipinski et al., 2007; Yang, Huang and Tan, 2016). In addition, certain environmentally prevalent toxicants can block Hh signaling. Piperonyl butoxide (PBO) is a semisynthetic pesticide additive developed in the late 1940s that is found in thousands of household and agricultural products, including aerosol sprays/foggers and lice shampoos (Daiss and Edwards, 2006). PBO is marketed as a synergist in pesticide formulations. It inhibits an insect’s cytochrome P450 detoxification enzymes, thereby augmenting the pesticidal activities of the active ingredient(s) (*e.g.,* pyrethroid pesticides). While PBO use is widespread, the potent developmental hazard of PBO was only recognized recently with the discovery that PBO can block Hh signaling (Everson et al., 2019; Horton et al., 2011; Wang et al., 2012).

Here, we tested the hypothesis that multifactorial genetic and/or environmental disruption of Hh signaling can interact with ethanol to cause craniofacial birth defects. We found that PBO and ethanol caused dose‐dependent malformations of the neurocranial cartilage of zebrafish. Single‐allele mutations in the Hh pathway gene *shha* sensitized embryos to both ethanol‐ and PBO‐induced defects. Ethanol and PBO synergistically interacted, such that PBO‐induced malformations were exacerbated when combined with a dose of ethanol that did not cause significant craniofacial defects on its own. Finally, we found that this PBO–ethanol interaction was even more dramatic in genetically predisposed *shha* embryos. Our results suggest that attenuation of Hh signaling could be a “hotspot” for multifactorial interactions in the genesis of FASD.

## Materials and Methods

### Zebrafish Husbandry and Embryo Collection

This study was conducted in accordance with the recommendations in *The Zebrafish Book, 5th edition (*Westerfield, 1993 *),* and the *Guide for the Care and Use of Laboratory Animals* of the National Institutes of Health. The protocol was approved by the University of Texas at Austin Institutional Animal Care and Use Committee (protocol number AUP‐2018‐00002). All zebrafish were housed at the University of Texas at Austin under IACUC‐approved conditions. Unless otherwise noted, fish are wild‐type AB strain. Developmental staging of embryos was determined by the identification of well‐characterized morphological features (Kimmel et al., 1995). For gene–environment studies, we used the hypomorphic *shha^tq252^* allele (ZFIN ID: ZDB‐FISH‐150901‐12482) (Chen et al., 1996), maintained on an AB background.

### Drug Preparation and Exposure

Piperonyl butoxide (5‐[2‐(2‐butoxyethoxy)ethoxymethyl]‐6‐propyl‐1,3‐benzodioxole, CAS 51‐03‐6) was obtained from Toronto Research Chemicals (Toronto, ON). PBO was dissolved in dimethyl sulfoxide (DMSO) for a stock concentration of 100 mM and stored at −20°C. These stock solutions were dissolved in embryo medium at the appropriate dosing concentration. Embryos were exposed from 6 to 24 hpf. This period of exposure was chosen to include the period of susceptibility for alcohol‐ and PBO‐induced HPE‐associated midfacial defects described in mice (Everson et al., 2019; Kietzman et al., 2014). At the end of the dosing period (24 hpf), the drug and embryo medium were removed and replaced with new embryo media. Control fish received the equivalent dose of DMSO. For alcohol experiments, absolute ethanol (200 proof) was dissolved in embryo media at the indicated final concentration. Previous studies have found that approximately 25 to 35% of the dose of alcohol in the media is taken up by the embryo (Flentke et al., 2014; Lovely, Nobles and Eberhart, 2014; Reimers, Flockton and Tanguay, 2004; Zhang, Ojiaku and Cole, 2013). This dose of ethanol does not cause significant craniofacial defects.

### Dual Bone and Cartilage Staining

Alizarin Red and Alcian Blue staining was performed as described (Walker and Kimmel, 2007). Briefly, embryos were fixed for 2 hours in 2% paraformaldehyde in phosphate‐buffered saline, followed by dehydration into 100% ethanol and overnight staining with Alcian Blue. Next, embryos were rehydrated and bleached with 3% H_2_O_2_/0.5% KOH before being stained with Alizarin Red. Finally, embryos were cleared and stored in 50% glycerol and 0.2% KOH until imaged.

### DNA Extraction and Genotyping

DNA was extracted from tails of zebrafish by incubating samples in 50 *μ*l of 50 nM NaOH at 95°C for 20 minutes before neutralizing by addition of 5 *μ*l 1M tris (pH = 8). Extracted DNA and lysate were stored at −20°C. PCR was conducted using standard methods and reagents, anneal temperature = 59.1°C. Genotyping primers were designed using Geneious (San Diego, CA). Genotyping was achieved using restriction fragment length polymorphism (RFLP), where a substitution is inserted to generate a specific restriction enzyme cut site in mutant fish. Primers sequences were as follows: *tq252‐shha* forward – 5′‐AGT GGC TGT GGC TTG AAG TAA CGTC‐3′, reverse – 5′‐TGA ATC TCG CTG CGG TGT TCTC‐3′. DNA products were then incubated at 37°C for 60 minutes with CutSmart buffer (New England Biolabs, Ipswich, MA, USA) and NlaIII enzyme (New England Biolabs, Ipswich, MA, USA), which cuts the restriction site CATG in mutant DNA strands to shorten the PCR product from 239 bp to 211 bp. Product bands were visualized in a 1.5% agarose gel using ethidium bromide.

### Embryo Imaging and Morphological Measurements

Alcian Blue‐ and Alizarin Red‐stained embryos were brightfield imaged in whole mount in 50% glycerol and 0.2% KOH on an Olympus SZX7 microscope (Shinjuku, Tokyo, Japan) with an Olympus DP22 camera. Flat mounts were prepared by manually separating dorsal neurocranial tissues from ventral viscerocranial tissues via an insect pin, as previously described (Kimmel et al., 1995). Flat‐mount images were acquired on a Zeiss AxioImager.A1 compound microscope (Oberkochen Germany) with a Zeiss AxioCam HRc camera. Linear measurements of embryos were determined using ImageJ (NIH).

### Statistics

Fisher’s exact test with Bonferroni multiple comparisons correction was used for determination of significance for incidence of scored defects. One‐way ANOVA with Tukey’s multiple comparisons correction was used for determination of significance for linear measurements. GraphPad Prism 6 (San Diego, CA, USA) was used for all analyses. An alpha value < 0.05 was considered significant.

## Results

### Mutations in *shha* Sensitize Embryos to PBO‐ and Ethanol‐Induced Craniofacial Malformations

As both alcohol and PBO have been linked to Hh pathway perturbation, we predicted that these teratogens would operate in gene–environment interactions with mutations in the *Shh* homologue *shha*. Embryos were generated that were either wild‐type or carried a single hypomorphic *shha* allele (*tq252*). These embryos were exposed to a range of concentrations of PBO or ethanol starting at the onset of gastrulation (6 hpf) until pharyngula stage (24 hpf). In the absence of an environmental insult, *shha* heterozygotes appeared normal, but PBO‐ or ethanol‐exposed embryos had defects of neural crest‐derived midfacial elements, the bilateral trabecula and ethmoid plate. These malformations ranged from subtle chondrocyte stacking defects within the trabeculae to severe midline deficits reminiscent of HPE‐associated phenotypes.

**Fig. 1:**
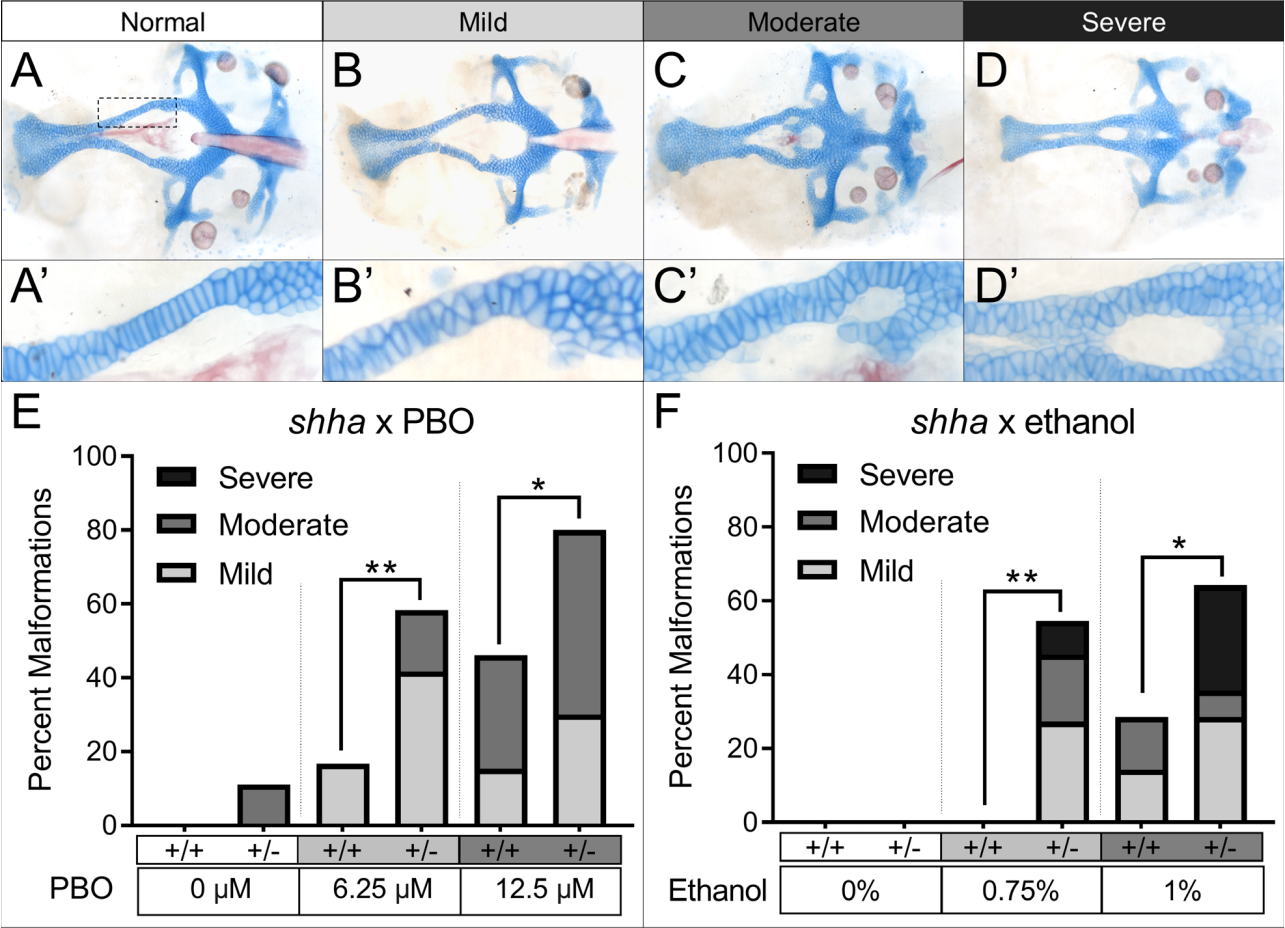
Hh pathway mutations sensitize embryos to ethanol‐ or PBO‐induced craniofacial defects. (**A**–**D**) Flat‐mount preparations of 5 dpf neurocrania showing the range of phenotypes observed. Phenotypes ranged from apparently normal to severe, with increasing severity determined by more extensive malformations of the bilateral trabeculae. All images were captured at 10X magnification. (**A**′–**D**′) 20X magnification of the right trabeculae of **A**–**D** shows cell arrangement defects in affected embryos, specifically the stacking defect in mild embryos (**B**′) compared to normal stacking (**A**′). (**E**–**F**) Wild‐type or embryos with a single‐allele mutation in *shha (tq252)* were exposed to 0% 0.75% or 1% ethanol (**E**) or 0, 6.25, or 12.5 μM PBO (**F**). Percent malformations (mild, moderate, and severe) are shown. Incidence of malformations was compared between genotypes for each treatment group using Fisher’s exact test with Bonferroni correction for multiple comparisons. *N* ≥ 15 embryos per genotype per treatment.

Malformations were measured using the following semi‐quantitative scoring criteria: 0 = apparently normal, 1 = mild chondrocyte stacking defects, 2 = moderate stacking defects, and 3 = severe trabecular defects with overt midfacial deficits (Fig. 1 *A*–*D*,*A*′–*D*′). At all doses, PBO‐induced trabecular defects were more frequent in heterozygous embryos compared to their wild‐type siblings (Fig. 1 *E*). A similar effect was observed in ethanol‐exposed embryos, with significantly more malformations observed in *shha* heterozygous embryos than their wild‐type siblings (Fig. 1 *F*). Strikingly, at 0.75% ethanol defects were only observed in heterozygous embryos.

### Ethanol and Piperonyl Butoxide Synergistically Interact to Cause Craniofacial Defects

**Fig. 2:**
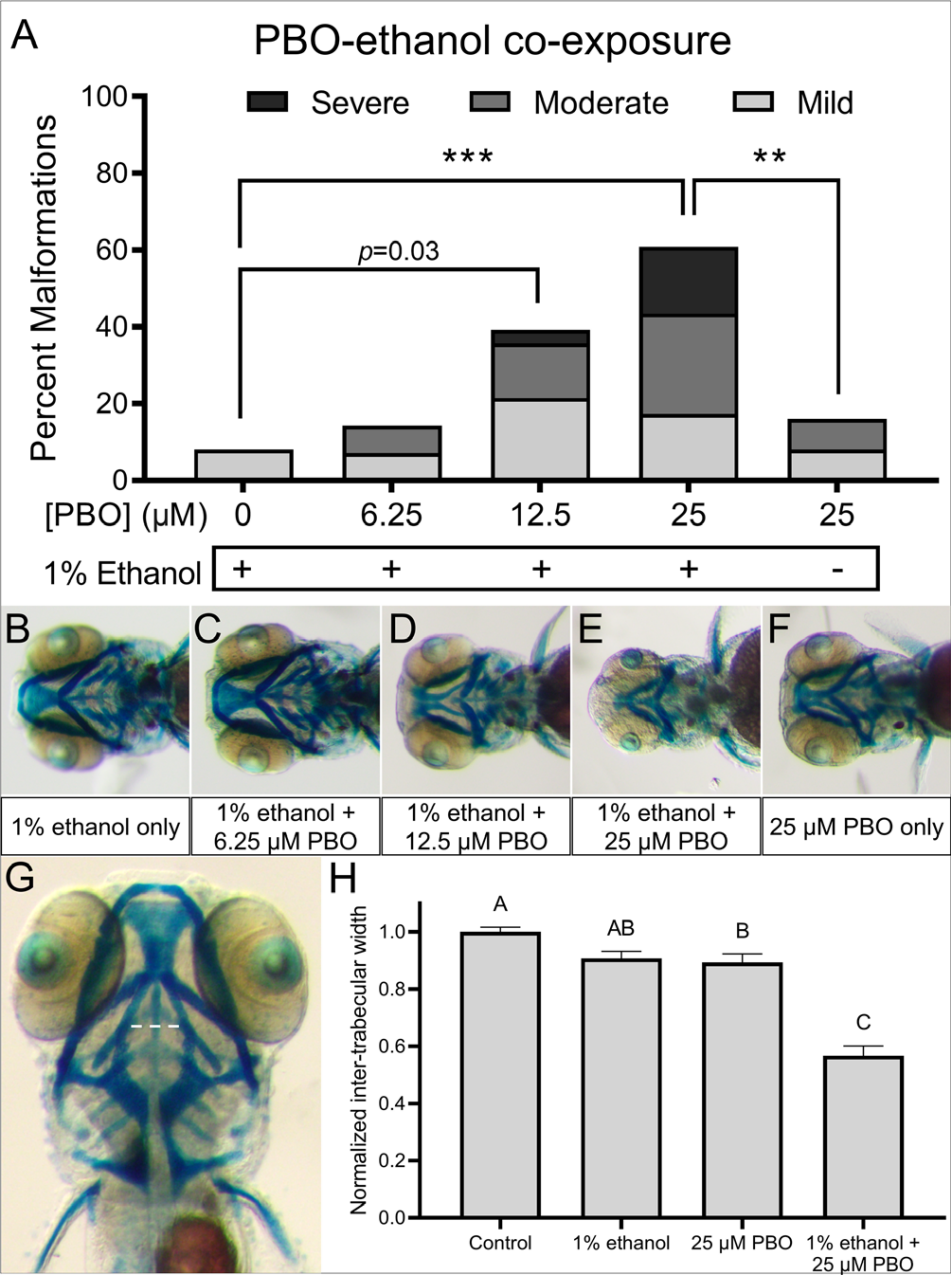
Ethanol and PBO synergistically interact to cause craniofacial defects. (**A**) Wild‐type embryos were exposed to 0, 6.25, 12.5, or 25 μM PBO with or without a 1% dose of ethanol. Percent malformations (mild, moderate, and severe) are shown. Incidence of malformations was compared between treatment groups using Fisher’s exact test with Bonferroni correction for multiple comparisons. ***p < 0.001, **p < 0.01. (**B**–**F**) Whole‐mount images of alcian‐ and alizarin‐stained embryos are shown for each treatment group. (**G**) 5‐day‐old alcian‐ and alizarin‐stained embryo marks the measurement for inter‐trabecular width (white dashed outline). (**H**) Quantification of inter‐trabecular widths. Mean width ± SEM is shown. Measurements were normalized to control and compared using one‐way ANOVA with Tukey’s multiple comparisons test. Different letters indicate statistically significant differences between the groups. See supplemental data for the specific multiple comparisons *p*‐values. *N* ≥ 28 embryos per treatment.

Co‐exposure to one or more additional environmental teratogens could also explain disparate outcomes following prenatal ethanol exposure in humans. We therefore exposed embryos to a range of concentrations of PBO (6.25, 12.5, or 25 μM) either alone or in combination with 1% v/v ethanol from 6 hpf ‐ 24 hpf. Neurocranial malformations were scored using the same criteria as in Fig. 1 *A*–*D*. We observed dose‐dependent neurocranial cartilage malformations in PBO‐exposed embryos (Fig. 2 *A*). Consistent with previous studies, embryos exposed to 1% ethanol alone showed few defects. However, coupling this dose of ethanol with PBO resulted in a high frequency of malformations. However, while embryos exposed to 6.25 μM PBO + 1% ethanol in media were not significantly different than embryos exposed to 1% ethanol alone, embryos exposed to either 12.5 or 25 μM PBO, respectively, displayed nearly significant (*p* = 0.03, not significant after multiple comparisons correction) or significantly more trabecular defects than siblings exposed to 1% ethanol alone. Exposure to 25 μM PBO caused malformations in 15% of embryos, and 1% ethanol alone caused malformations in 6% of embryos. However, co‐exposure to both 25 μM PBO and 1% ethanol caused defects in 60% of embryos. This interaction is highly synergistic, as the actual rate of malformations (60%) is dramatically higher than the predicted incidence for an additive effect (21%). Interestingly, the wild‐type AB fish from these experiments were less sensitive to environmental perturbation than their wild‐type counterparts derived from the *tq252* strain shown in Fig. 1. While we do not know the exact reason for these differences, we note that the *tq252* allele was generated in the tubigen genetic background (Chen et al., 1996). Thus, it is likely that differences between the tubigen and AB genetic backgrounds mediate this differential sensitivity.

This interaction was further characterized using linear measurements of inter‐trabecular widths to assess midfacial deficits (Fig. 2 *G*). We found that while exposure to 1% ethanol alone did not cause a significant reduction in inter‐trabecular width compared to control, 25 μM PBO alone did cause a significant decrease in inter‐trabecular width (Fig. 2 *H*). This effect was significantly more robust when 25 μM PBO was co‐exposed with 1% ethanol, demonstrating a synergistic interaction between these factors. A full table of ANOVA results can be found in supplemental data.

### Multifactorial Modeling of Craniofacial Birth Defects

We hypothesize birth defects in humans may involve multiple exposures superimposed upon genetic predisposition (Graham and Shaw, 2005). Thus, we next exposed wild‐type or heterozygous *shha* embryos to a low dose of PBO, ethanol, or both chemicals in combination. Consistent with our previous findings, embryos with heterozygous *shha* mutations were indistinguishable from their wild‐type siblings under control conditions. However, heterozygous embryos were sensitized to PBO‐ and ethanol‐induced defects. Heterozygous embryos exposed to either 3.125 μM PBO or 0.5% ethanol alone had significantly more craniofacial defects than their wild‐type siblings. For wild‐type embryos, while 0.5% ethanol caused no observable defects and 3.125 μM PBO caused defects in 12% of wild‐type embryos, the combination of these chemicals caused defects in 45% of embryos. This incidence (45%) is greater than expected for an additive effect (12%), indicating a synergistic interaction between these chemicals. Heterozygous embryos were sensitized to this PBO–ethanol interaction, with significantly more defects observed in co‐exposed heterozygous embryos compared to their wild‐type siblings.

**Fig. 3:**
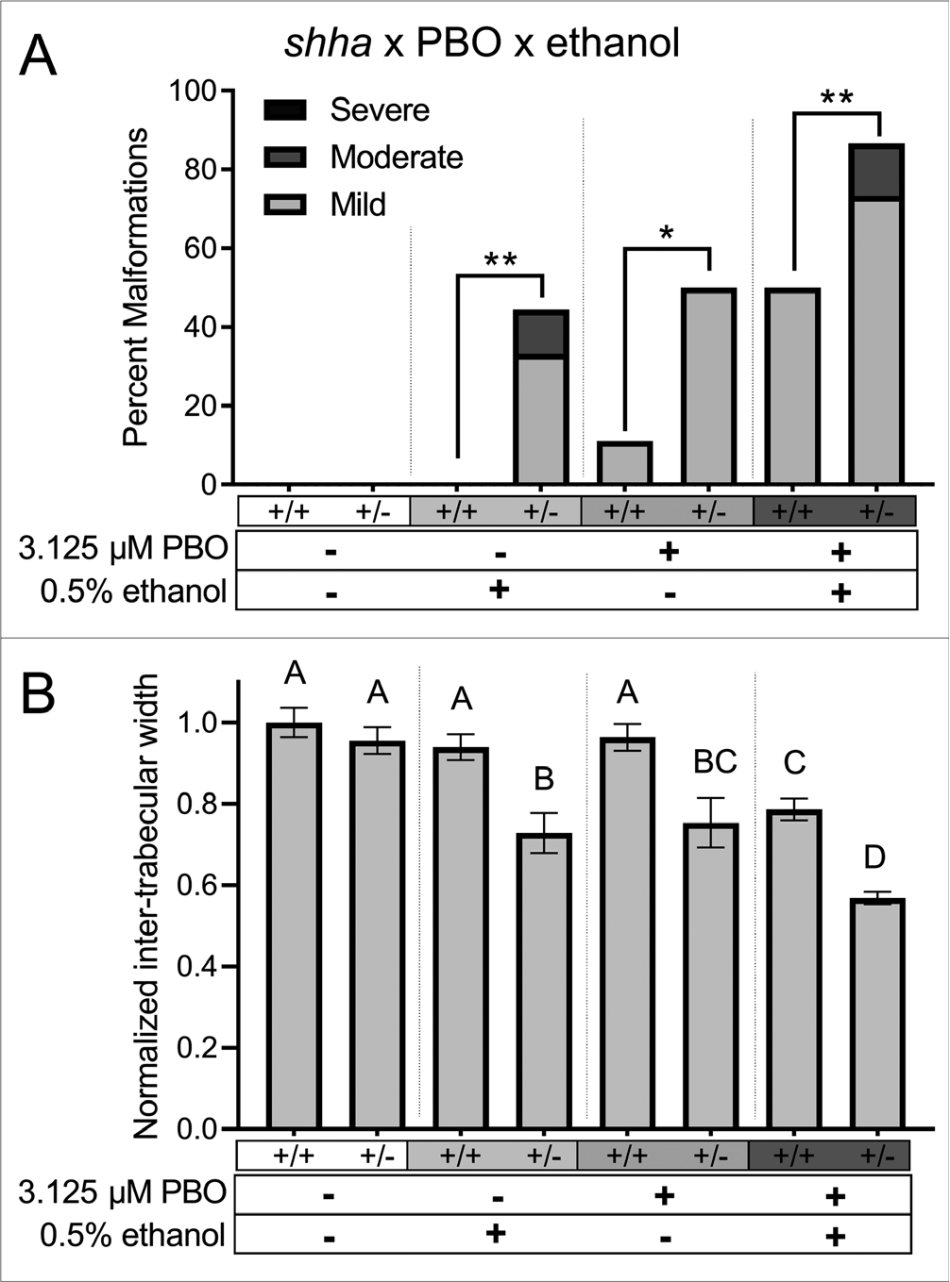
Mutations in *shha* sensitize embryos to multifactorial interactions between ethanol and PBO. (**A**) Wild‐type (+/+) or heterozygous (+/‐) embryos for *shha (tq252)* were exposed to subthreshold doses of PBO (3.125 μM), ethanol (0.5%), or the combination of both chemicals (3.125 μM PBO + 0.5% ethanol). Percent malformations (mild, moderate, and severe) are shown. Incidence of malformations was compared between genotypes for each treatment group using Fisher’s exact test with Bonferroni correction for multiple comparisons. (**B**) Quantification of inter‐trabecular widths. Mean width ± SEM is shown. Measurements were normalized to control and compared using one‐way ANOVA with Tukey’s multiple comparisons test. Different letters indicate statistically significant differences between the groups. See supplemental data for the specific multiple comparisons *p*‐values. *N* ≥ 14 embryos per genotype per treatment.

Again, we quantified this interaction using linear measurements (Fig. 3 *B*). No difference was observed between wild‐type and heterozygotes under control conditions. Wild‐type embryos exposed to either 0.5% ethanol or 3.125 μM PBO alone had inter‐trabecular widths that were not significantly different than control embryos. Ethanol alone (0.5%) caused a significant reduction of inter‐trabecular width in *shha* heterozygous embryos compared to controls or wild‐type siblings receiving the same dose. PBO alone (3.125 μM) similarly only caused a significant reduction in *shha* heterozygotes. Wild‐type embryos co‐exposed to both PBO and ethanol had reduced inter‐trabecular widths compared to wild‐type embryos exposed to either PBO or ethanol alone. Finally, the inter‐trabecular widths of co‐exposed heterozygous embryos were significantly reduced compared to all other groups, demonstrating multifactorial interactions between these 3 factors. A full table of ANOVA results can be found in supplemental data.

## Discussion

We used a zebrafish model of early gestational exposure to alcohol to examine multifactorial interactions in the pathogenesis of alcohol‐induced craniofacial defects. We demonstrated interactions between alcohol, PBO, and *shha*. These interactions compound synergistically, with co‐perturbations having dramatically more effect than a single insult and the tripartite interaction being most severe. Our results highlight the complex interactions that can modify alcohol teratogenesis and the need for research examining multifactorial exposures.

Several lines of evidence suggest a genetic component to FASD. Twin studies provide some of the strongest evidence from humans. Monozygotic twins, who inherit an identical set of genes, were found to have comparatively higher FASD concurrence rates than dizygotic twins, who inherit roughly 50% of the same genes (Astley Hemingway et al., 2018; Eberhart and Parnell, 2016; Streissguth and Dehaene, 1993). In these studies, environmental inputs can be considered constant. Therefore, these differences in outcomes for mono‐ versus dizygotic twins may be due to one of the dizygotic twins having differential expression for any number of alcohol susceptibility genes. Research using model organisms provides several lines of evidence for a genetic role in alcohol‐induced craniofacial defects. Among the genes and pathways associated with altered alcohol teratogenicity are mutations that disrupt the Hedgehog (Hh) signaling pathway. Mutations in core Hh pathway genes including *Shh*, *Gli2*, and *Cdon* have each been individually shown to heighten an embryo’s susceptibility to alcohol‐induced defects in mice (Hong and Krauss, 2012; Kietzman et al., 2014). Similarly, knockdown of *shh* in zebrafish sensitizes embryos to ethanol teratogenesis (Burton et al., 2017; Zhang, Anderson and Cole, 2015; Zhang et al., 2014; Zhang, Ojiaku and Cole, 2013). The Hh pathway requires a functional primary cilium for signaling (Hoover et al., 2008). Mutation of the ciliary gene *Mns1* sensitized embryos to ethanol‐induced craniofacial defects (Boschen et al., 2018). The findings herein further suggest a genetic component for FASD and show that Hedgehog pathway mutations sensitize embryos to alcohol. Moreover, our findings demonstrate that nongenetic inputs may also modify the genetics of alcohol susceptibility.

The contribution of environmental co‐exposures in the etiology of FASD is suspected, but few examples of specific interactions have been elucidated. Also known as “mixture effects,” studies examining the developmental effects of combined exposure to multiple environmental factors are complex and rare. However, several examples come from the nutritional sciences. Co‐environmental interactions have been observed between alcohol and dietary factors like iron deficiency (Helfrich et al., 2018; Huebner et al., 2016; Huebner et al., 2015; Rufer et al., 2012), which exacerbates alcohol teratogenicity, and choline or folate, which can reduce ethanol‐induced deficits (Bottom, Abbott and Huffman, 2020; Muralidharan, Sarmah and Marrs, 2015; Serrano et al., 2010; Shi et al., 2014; Thomas et al., 2010; Thomas and Tran, 2012). Choline supplementation in children has also been shown to be capable of partially ameliorating FASD‐associated neurocognitive deficits postnatally (Wozniak et al., 2020). In addition, either too much or too little retinoic acid can disrupt Hh signaling (Power, Lancman and Smith, 1999; Wang et al., 2019). Alcohol and its metabolite acetaldehyde disrupt retinoic acid homeostasis (Clugston and Blaner, 2012; Deltour, Ang and Duester, 1996; Kane et al., 2010; Napoli, 2011; Shabtai et al., 2018; Yelin et al., 2005), and supplementation of retinoic acid can partially rescue ethanol‐induced teratogenesis (Muralidharan, Sarmah and Marrs, 2015; Muralidharan, Sarmah and Marrs, 2018; Zhang, Anderson and Cole, 2015). For nondietary environmental factors, interactions have been observed between recreational drugs and alcohol; for example, THC, CBD, and synthetic cannabinoids have each been shown to interact with alcohol to cause defects in mice and zebrafish (Boa‐Amponsem et al., 2020; Fish et al., 2019; Gilbert et al., 2015) and behavioral deficits in rats and zebrafish (Boa‐Amponsem et al., 2020; Breit, Zamudio and Thomas, 2019a; Breit, Zamudio and Thomas, 2019b). Together, these reports clearly demonstrate the possibility of alcohol–environment interactions in FASD.

A major hurdle in furthering our understanding of alcohol–environment interactions is how to rationally select chemical agents from the hundreds of thousands of EPA‐registered chemicals to study in co‐exposure models. Part of the EPA’s Toxicology Forecasting in the 21^st^ century (Tox21) initiative, the ToxCast library is a source for environmentally relevant chemicals linked with data from hundreds of cell‐based assays *in vivo* assays, as well as each chemical’s physicochemical properties (Richard et al., 2016; Truong et al., 2014). This enables clustering of chemicals that may interact mechanistically. Using this database and the high fecundity of the zebrafish, future studies will comprehensively assess environmental co‐exposures to identify novel interactors.

The genesis of human craniofacial defects is complex and poorly defined. This likely stems from their multifactorial bases, which complicates the identification of specific causative factors and makes modeling *in vivo* difficult. For alcohol, a recent study characterized multifactorial interactions in mice and zebrafish. Fish *et al*. found that alcohol and cannabinoids interact to cause craniofacial defects. This interaction appeared dependent on disruption of Hh signaling. Injection of Shh‐N protein was capable of rescuing alcohol‐cannabinoid‐induced defects, faithfully demonstrating a multifactorial interaction between 2 environmental factors and a critical genetic pathway (Fish et al., 2019).

Hh signaling is linked to several craniofacial birth defects in humans, including HPE and orofacial clefts (OFCs) (Jiang, Bush and Lidral, 2006; Lidral, Moreno and Bullard, 2008; Roessler et al., 1996; Roessler et al., 2003). Like FASD, the bases of HPE and OFCs are complex with apparent genetic and environmental contributions (Heyne et al., 2016; Roessler, Hu and Muenke, 2018; Roessler and Muenke, 2010; Solomon et al., 2012; Solomon et al., 2010). For HPE, mutations in *SHH* are the most common single‐gene cause (Nanni et al., 1999; Roessler et al., 1996). Previous work has demonstrated that gastrulation‐stage ethanol exposure causes HPE (Hong and Krauss, 2012; Hong and Krauss, 2017; Kietzman et al., 2014; Lipinski et al., 2012a; Sulik, 1984). Additionally, mutations in the Hh pathway gene *Cdon* sensitize mice to alcohol‐induced HPE, which is rescued by additional mutation of the negative pathway regulator *Ptch1* (Hong and Krauss, 2012). Similarly, a gastrulation‐stage exposure to PBO causes HPE, and *Shh* mutations sensitize embryos to PBO‐induced HPE in mouse (Everson et al., 2019). Few published studies have directly examined the contribution of environmental exposures in HPE etiology. Thus, the data herein represent the first direct evidence that compound exposures to alcohol and PBO can interact with disease‐associated mutations to cause clinically relevant birth defects.

Pharmacological Hh pathway disruption can cause diverse craniofacial defects, including both HPE and OFCs, with the specific outcome dependent upon time of exposure (Everson et al., 2017; Heyne et al., 2015; Lipinski et al., 2010). While our exposures initiated at gastrulation, they extended beyond the end of gastrulation, to the pharyngula stage, in order to capture this later sensitive time period. Therefore, the interactions we identified may hold importance for other common craniofacial syndromes like OFCs. Furthermore, our findings may suggest a broader role of environmental Hh pathway perturbation in the etiology of human craniofacial diseases. Given the large number of developmental processes controlled by Hh signaling, our findings are likely to be relevant to the complex basis of many human birth defects.

## Conflicts of Interest

The authors declare that they have no competing interests.

## Author Contributions

JKE and JLE designed studies; JLE and RB conducted experiments and acquired data; JLE analyzed data; JLE and JKE wrote the manuscript; and all authors read and approved the final manuscript.

## Ethics Approval

This study was conducted in accordance with the recommendations in *The Zebrafish Book, 5th edition (*Westerfield, 1993 *),* and the *Guide for the Care and Use of Laboratory Animals* of the National Institutes of Health. The protocol was approved by the University of Texas at Austin Institutional Animal Care and Use Committee (protocol number AUP‐2018‐00002). All zebrafish were housed at the University of Texas at Austin under IACUC‐approved conditions.

## Supporting information


**Data S1** ANOVA *p*‐value summary.
